# Supplementary figures and images for: Efflux pumps and membrane permeability contribute to intrinsic antibiotic resistance in Mycobacterium abscessus
Source: PLoS Pathog. 2025 Apr 10;21(4):e1013027. doi: 10.1371/journal.ppat.1013027 (PMC12017575; doi:10.1371/journal.ppat.1013027)

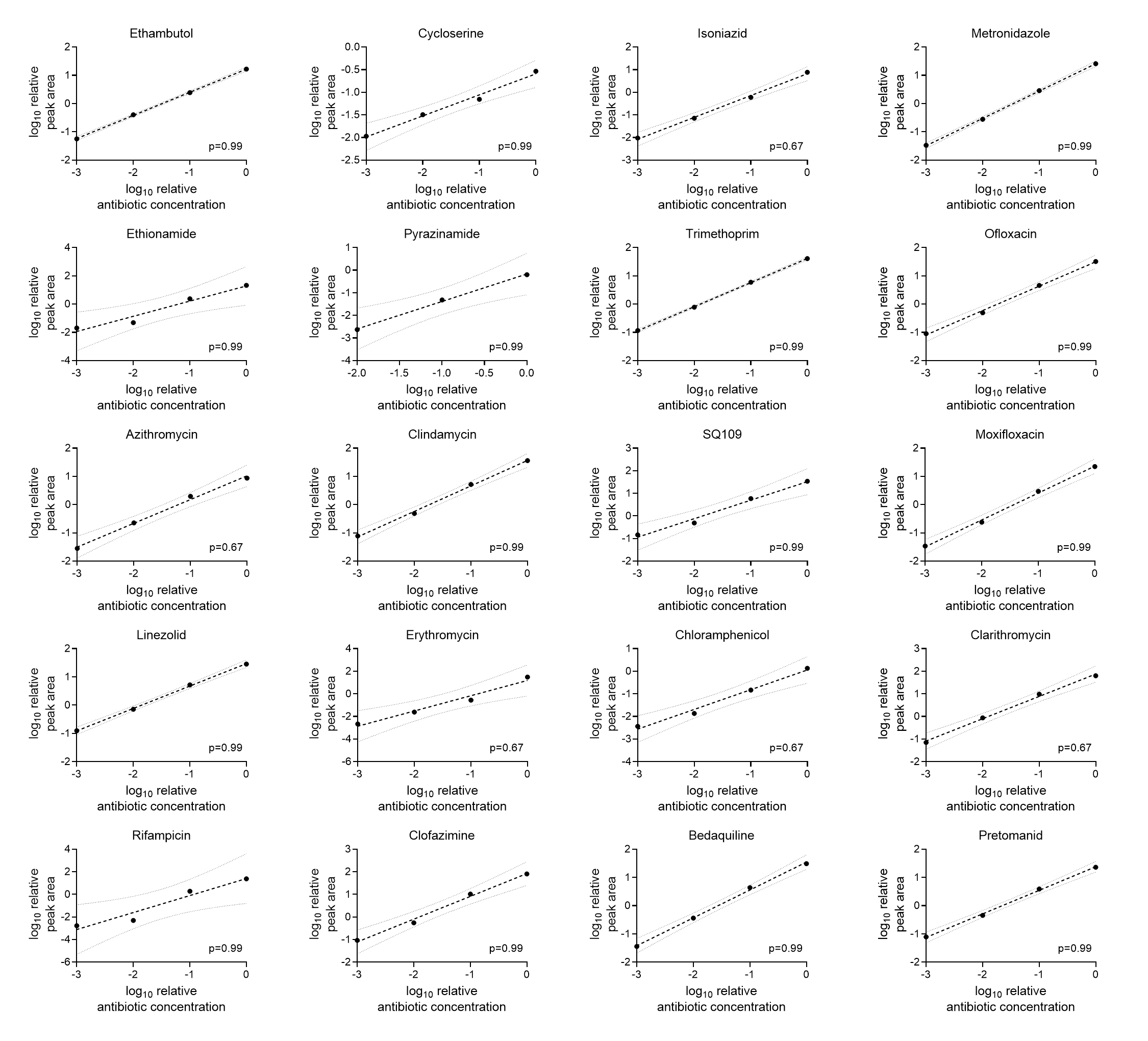

Supplement: S1 Fig — LC-MS measurement of indicated antibiotics over 1000-fold range of concentrations. Peak areas are normalized to internal standard, and antibiotic concentrations are normalized to the highest standard concentration. Line of best fit represents a simple linear regression and is represented +/- 95% confidence intervals. p-value derived from a Wald–Wolfowitz runs test to identify deviation from the linear fit. (TIF) [file ppat.1013027.s001.tif]

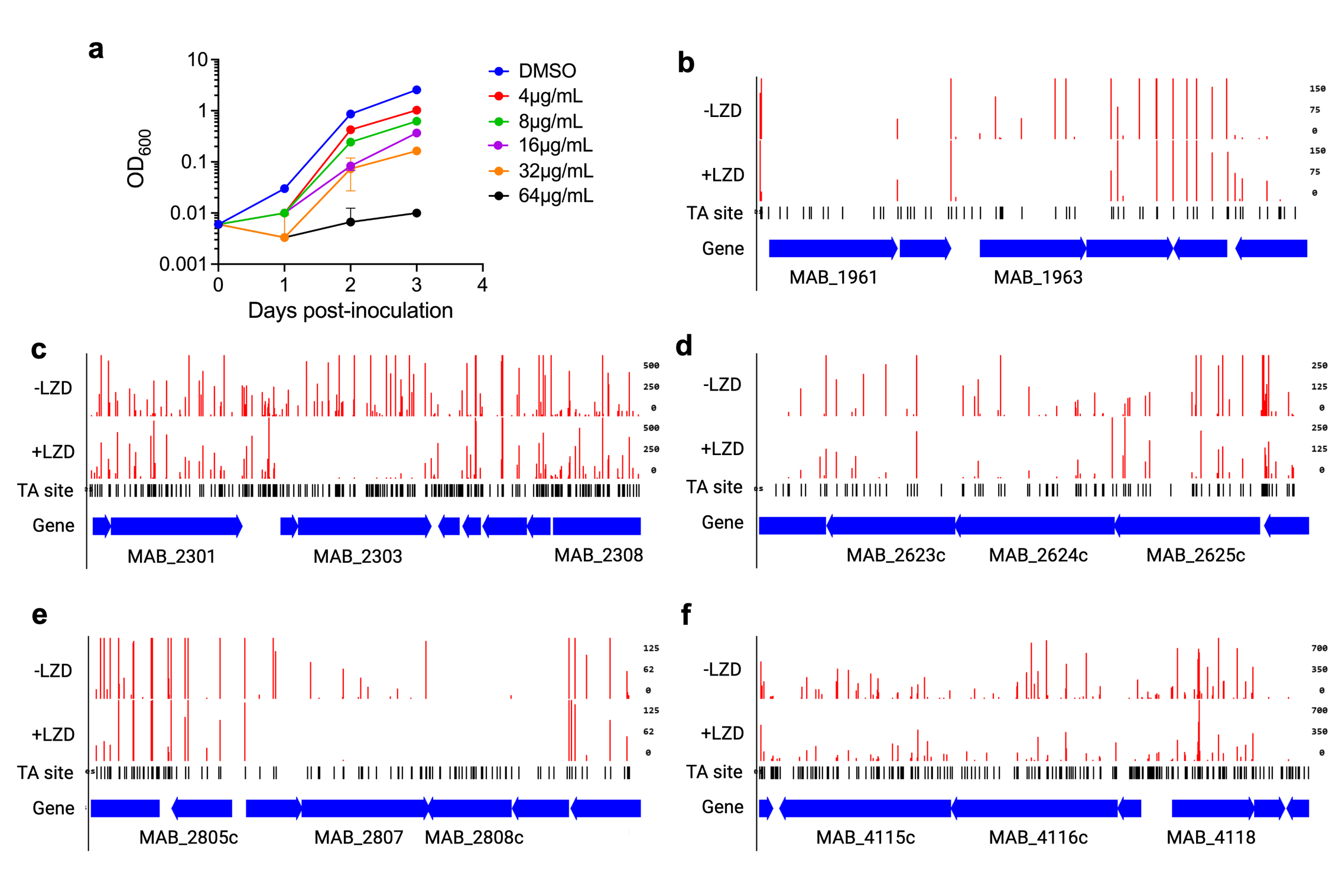

Supplement: S2 Fig — a, Relative growth as measured by optical density of M. abscessus clinical isolate BWH-F with specified concentrations of linezolid over time. Data are represented as individual values along with mean ± s.d. n = 3 biological replicates. b-f, Transposon insertion counts for indicated genes in representative replicates of the -linezolid and +linezolid conditions. Insertion counts are normalized to the local maximum. DMSO = dimethylsulfoxide. (TIF) [file ppat.1013027.s002.tif]

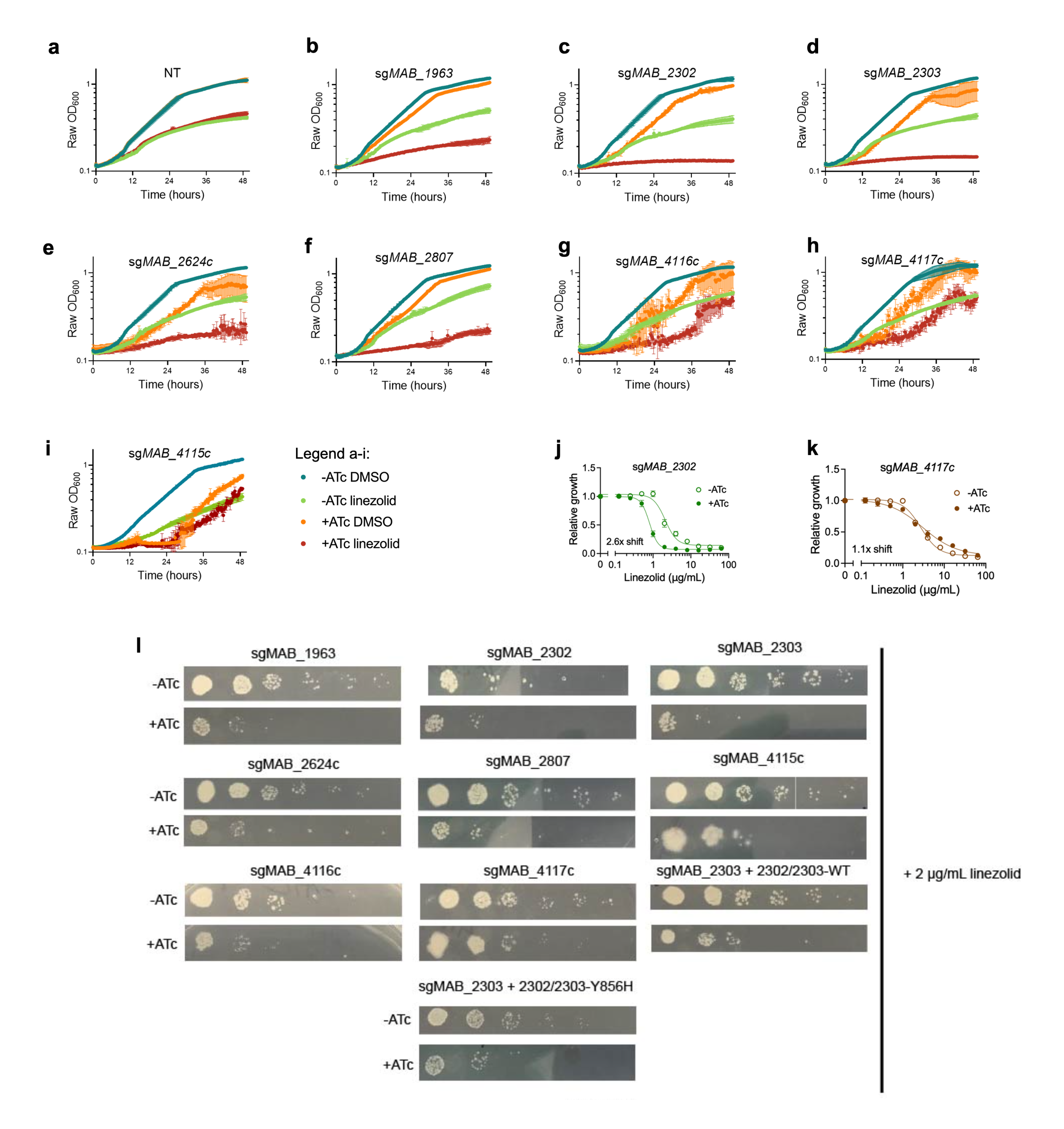

Supplement: S3 Fig — a-i, OD600 over time of pre-depleted M. abscessus ATCC19977 strains with sgRNAs targeting membrane transporter genes treated with 1μg/mL linezolid or vehicle along with ±ATc for 48 hours. j,k, Relative growth of M. abscessus ATCC19977 strains with sgRNAs targeting indicated membrane transporter genes as measured by a colorimetric dye treated with indicated concentrations of linezolid along with ±ATc for 48 hours. DMSO = dimethyl sulfoxide. ATc = anhydrotetracycline. NT = non-targeting sgRNA. l, Images of indicated M. abscessus CRISPRi strains pre-depleted for 24 hours with 500 ng mL−1 ATc, then plated as 10-fold serial dilutions on 7H10 agar plates containing 2 μg/mL linezolid and either 500 ng mL−1 ATc or vehicle. Initial culture density was normalized for each strain +/- ATc, but not between different CRISPRi strains. Images are representative of biological triplicates. (TIF) [file ppat.1013027.s003.tif]

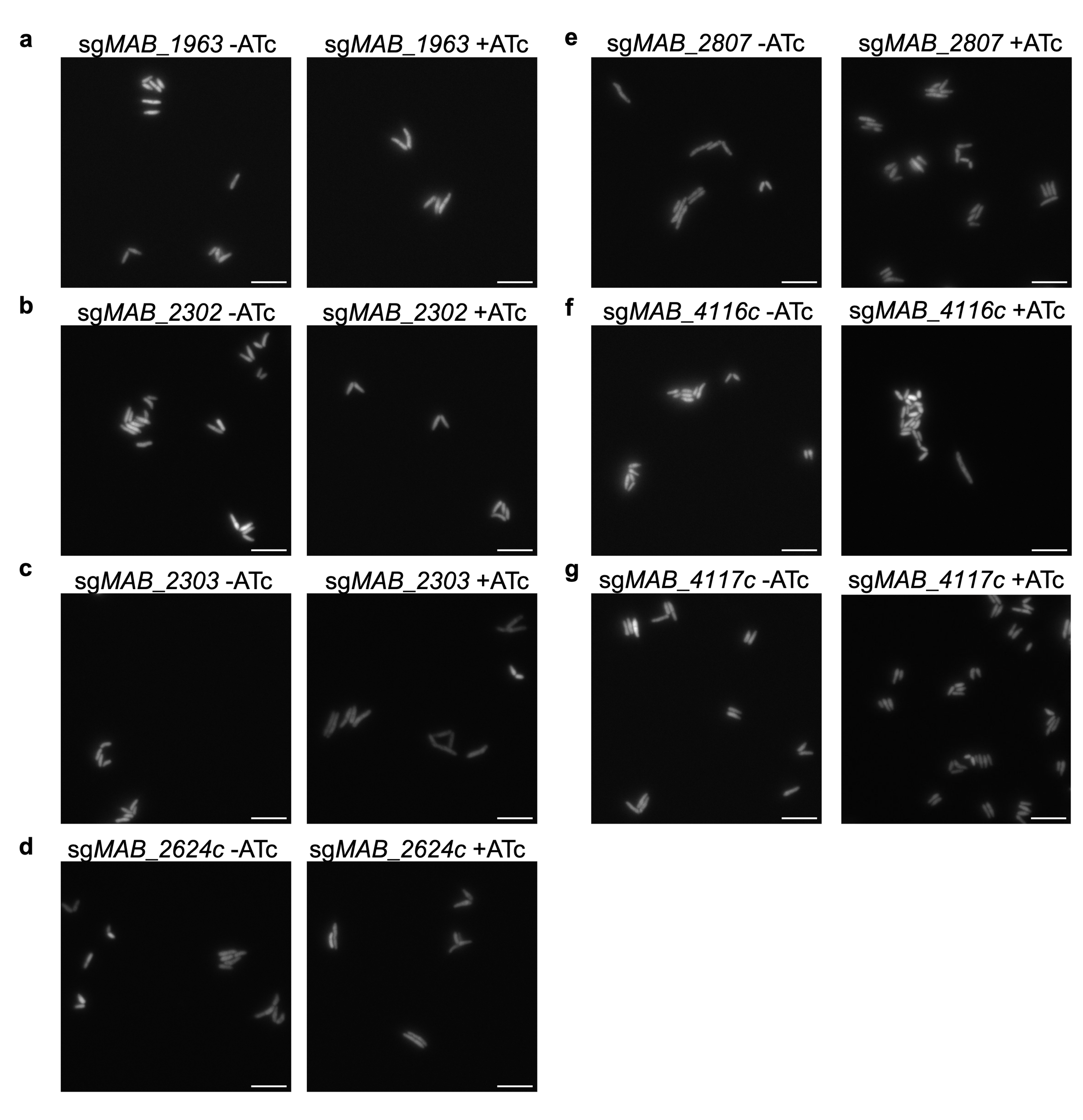

Supplement: S4 Fig — a-g, Representative fixed cell widefield microscopy images of M. abscessus ATCC19977 strains with sgRNAs targeting membrane transporter genes in the presence or absence of ATc for 24 hours prior to fixation. Images were taken at 100x magnification. Scale bar = 5 µ m. (TIF) [file ppat.1013027.s004.tif]

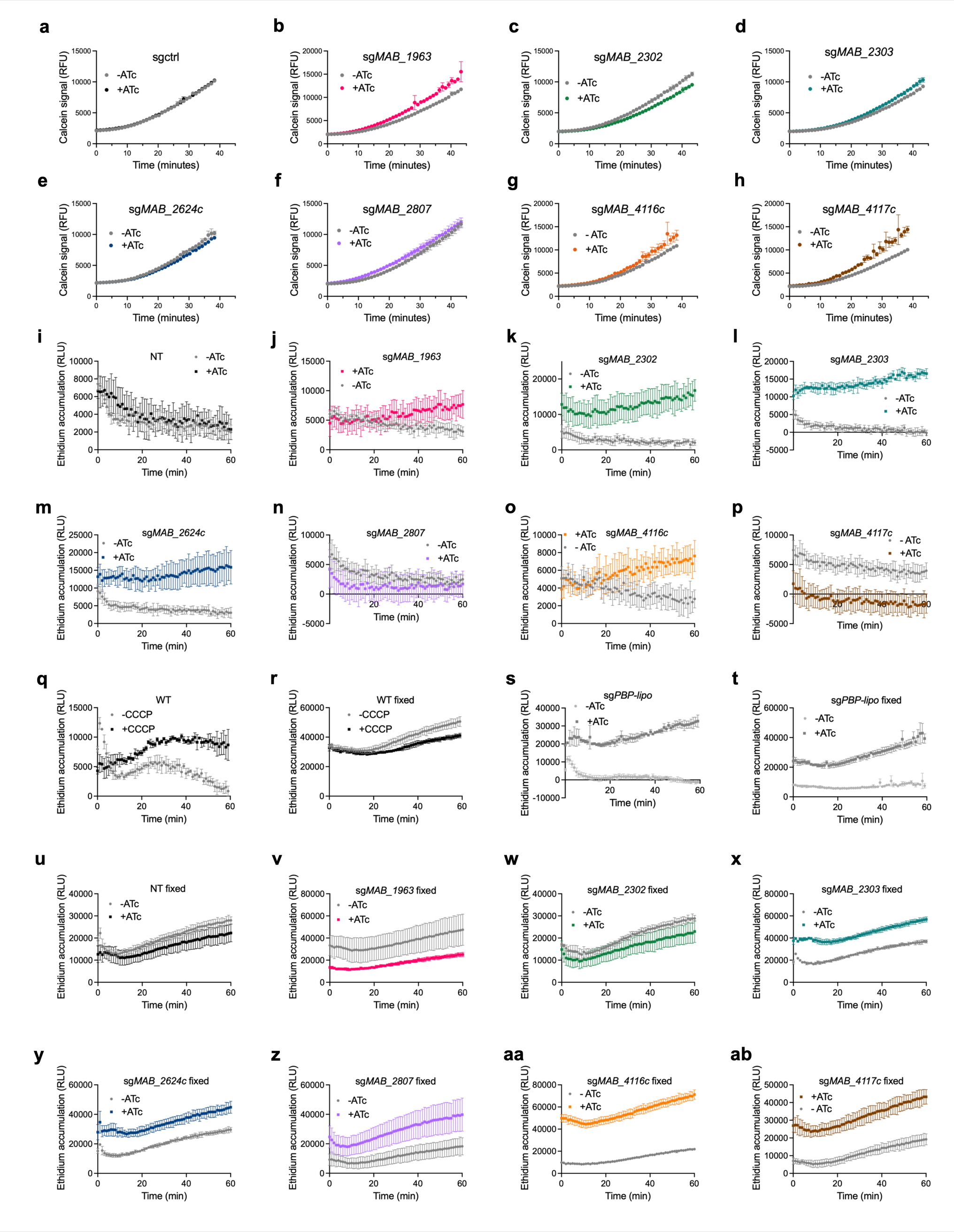

Supplement: S5 Fig — a-h, Calcein accumulation in M. abscessus ATCC19977 strains with sgRNAs targeting membrane transporter genes in the presence or absence of ATc for 24 hours prior to addition of calcein AM. Data are represented as individual values along with mean ± s.d. n = 3 biological replicates. ATc = anhydrotetracycline. i-p, Ethidium accumulation as measured by fluorescence over time in live M. abscessus ATCC19977 strains with indicated sgRNAs targeting membrane transporter genes treated with 500ng mL−1 ATc for 24 hours prior to addition of ethidium bromide. q-r, Ethidium accumulation as measured by fluorescence over time in live and fixed wildtype M. abscessus ATCC19977 treated with 50μM CCCP. s-ab, Ethidium accumulation in (s) live or (t-ab) fixed M. abscessus ATCC19977 strains with indicated sgRNAs targeting membrane transporter genes pre-treated with 500ng mL−1 ATc for 24 hours. Data are represented as individual values along with mean ± s.d. n = 3 biological replicates. ATc = anhydrotetracycline. WT = wildtype. NT = non-targeting sgRNA. (TIF) [file ppat.1013027.s005.tif]

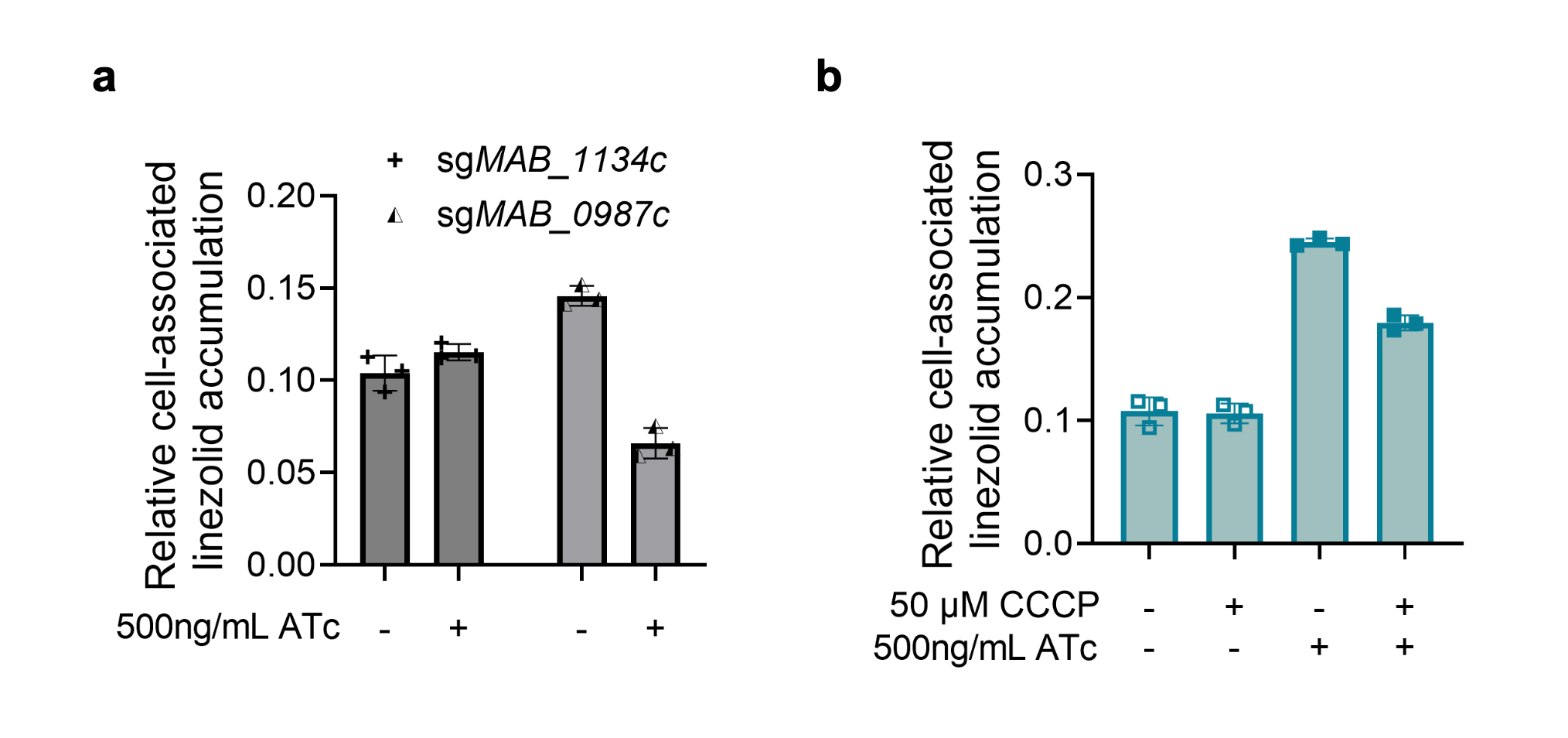

Supplement: S6 Fig — a, LC-MS measurement of cell-associated accumulation of linezolid in knockdown strains of MmpL proteins that were not required for survival of linezolid as determined by TnSeq. Pre-depleted (+ATc) sgMAB_1134c or sgMAB_0987c CRISPRi M. abscessus strains were incubated for 4 hr with antibiotics. Values normalized to internal standard and initial antibiotic levels in media prior to incubation. Data are represented as individual values along with mean ± s.d. n = 3 biological replicates. b, LC-MS measurement of cell-associated accumulation of linezolid in a pre-depleted (+ATc) sgMAB_2303 knockdown strain of M. abscessus. After 18 hr ATc pre-induction, strains were incubated for 10 minutes with 50 μM CCCP or with vehicle, then incubated with or without CCCP along with 20 μM linezolid. Values normalized to internal standard and initial antibiotic levels in media prior to incubation. Data are represented as individual values along with mean ± s.d. n = 3 biological replicates. Data are represented as individual values along with mean ± s.d. n = 3 biological replicates. ATc = anhydrotetracycline. CCCP = carbonyl-cyanide m-chlorophenylhydrazone. (TIF) [file ppat.1013027.s006.tif]

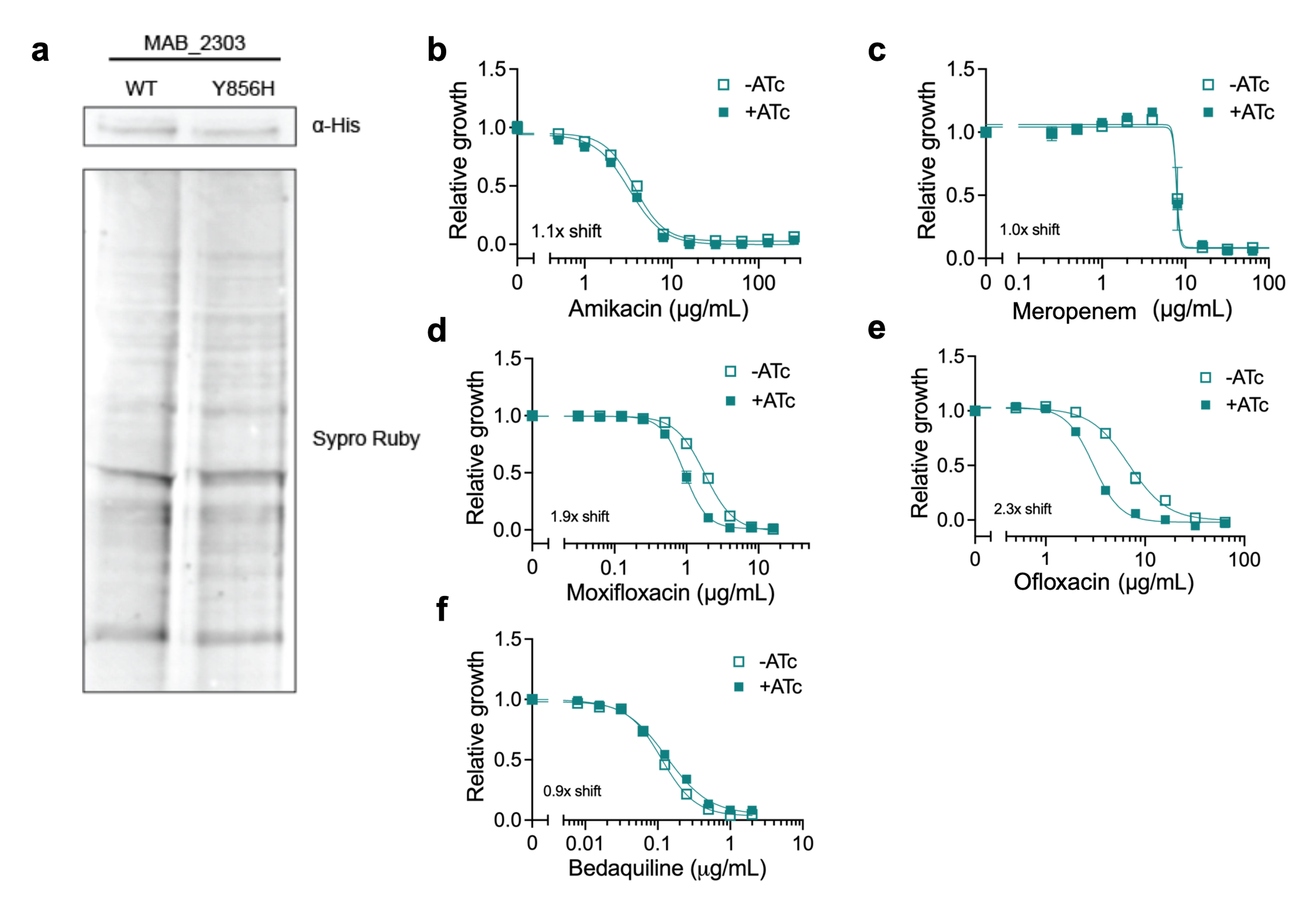

Supplement: S7 Fig — a, Western blot depicting expression of MAB_2303 WT or MAB_2303 Y856H each tagged with 6X-His. Sypro Ruby staining of total protein abundance is included as a loading control. b-f, Relative growth of pre-depleted sgMAB_2303 M. abscessus strain as measured by reduction of a colorimetric dye after treatment with indicated concentrations of (b) amikacin, (c) meropenem, (d) moxifloxacin, (e) ofloxacin, and (f) bedaquiline in the presence or absence of 500 ng mL−1 ATc for 24 hours. Values normalized to vehicle only control per drug. Data are represented as individual values along with mean ± s.d. n = 3 biological replicates. ATc = anhydrotetracycline. (TIF) [file ppat.1013027.s007.tif]
